# Supplementary material for: Hospital Mortality and Morbidity in Diabetic Patients with COVID-19: A Retrospective Analysis from the UAE
Source: Int J Environ Res Public Health. 2024 May 29;21(6):697. doi: 10.3390/ijerph21060697 (PMC11204093; doi:10.3390/ijerph21060697)
Supplement: Supplementary file 1 [file ijerph-21-00697-s001.zip › ijerph-2998762-supplementary.pdf]

Supplementary Table S1. Summary of baseline diabetes-related features and indicators among the diabetic patients (n=355).

Item	Indicator	n (%)
**Diabetes Type**	**Type 1**	6 (1.8)
	**Type 2**	329 (98.2)
**Diabetes Treatment**	**Insulin**	72 (21.5)
	**Injectable GLP-1 Agonists**	50 (14.9)
	**Oral Antidiabetic Agents**	125 (37.3)
**Diabetes Complications**	**Diabetic Foot**	3 (0.9)
	**Retinopathy**	3 (0.9)
	**Nephropathy**	35 (10.4)
	**Neuropathy**	9 (2.7)

GLP-1: glucagon-like peptide 1.

Supplementary Table S2. Summary of the presenting symptoms among the nondiabetic and diabetic patients at the time of admission.

Item	Subitem	Total (N= 427)	Nondiabetic (n= 92)	Diabetic (n= 335)	*P-value*
**Symptoms at presentation** *n (%)*	**Dyspnea**	291 (68.1)	67 (72.8)	224 (66.9)	*0.17*
	**Fever**	271 (63.5)	65 (70.7)	206 (61.5)	*0.07*
	**Cough**	260 (60.9)	59 (64.1)	201 (60.0)	*0.28*
	**Fatigue**	93 (21.8)	21 (22.8)	72 (21.5)	*0.44*
	**Myalgia**	80 (18.7)	35 (38.0)	45 (13.4)	*<0.001*
	**Chest pain**	70 (16.4)	16 (17.4)	54 (16.1)	*0.44*
	**Vomiting**	51 (11.9)	22 (23.9)	29 (8.7)	*<0.001*
	**Diarrhea**	45 (10.5)	17 (18.5)	28 (8.4)	*0.006*
	**Headache**	25 (5.9)	5 (5.4)	20 (6.0)	*0.54*
	**Sore throat**	24 (5.6)	6 (6.5)	18 (5.4)	*0.43*
	**Loss of smell/taste**	17 (4.0)	2 (2.2)	15 (4.5)	*0.25*
